# ABI3, a component of the WAVE2 complex, is potentially regulated by PI3K/AKT pathway

**DOI:** 10.18632/oncotarget.18840

**Published:** 2017-06-29

**Authors:** Lais Moraes, Nilson I.T. Zanchin, Janete M. Cerutti

**Affiliations:** ^1^ Genetic Bases of Thyroid Tumors Laboratory, Division of Genetics, Department of Morphology and Genetics, Escola Paulista de Medicina, Universidade Federal de São Paulo, São Paulo, Brazil; ^2^ Instituto Carlos Chagas, Fundação Oswaldo Cruz/FIOCRUZ, Curitiba, Paraná, Brazil

**Keywords:** *ABI3*, follicular thyroid carcinoma, WAVE2, CYFIP1, PI3K/AKT

## Abstract

We previously reported that *ABI3* expression is lost in follicular thyroid carcinomas and its restoration significantly inhibited cell growth, invasiveness, migration, and reduced tumor growth *in vivo*. The mechanistic basis by which ABI3 exerts its tumor suppressive effects is not fully understood. In this study, we show that ABI3 is a phosphoprotein. Using proteomic array analysis, we showed that ABI3 modulated distinct cancer-related pathways in thyroid cancer cells. The KEA analysis found that PI3K substrates were enriched and forced expression of ABI3 markedly decreased the phosphorylation of AKT and the downstream-targeted protein pGSK3β. We next used immunoprecipitation combined with mass spectrometry to identify ABI3-interacting proteins that may be involved in modulating/integrating signaling pathways. We identified 37 ABI3 partners, including several components of the canonical WAVE regulatory complex (WRC) such as WAVE2/CYF1P1/NAP1, suggesting that ABI3 function might be regulated through WRC. Both, pharmacological inhibition of the PI3K/AKT pathway and mutation at residue S342 of ABI3, which is predicted to be phosphorylated by AKT, provided evidences that the non-phosphorylated form of ABI3 is preferentially present in the WRC protein complex. Collectively, our findings suggest that ABI3 might be a downstream mediator of the PI3K/AKT pathway that might disrupt WRC via ABI3 phosphorylation.

## INTRODUCTION

We previously reported that *ABI3* (ABL-Interactor member 3) expression is lost in most thyroid carcinomas, compared to benign lesions and normal thyroid tissues. Moreover, *ABI3* re-expression in a human follicular thyroid carcinoma cell line significantly inhibited cell proliferation, invasion, and migration *in vitro* and reduced tumor growth *in vivo*. The partial re-acquisition of a less aggressive phenotype was accompanied by increase of cellular senescence [1]. We also demonstrated that transcriptional silencing of ABI3 in thyroid cancer occurs via methylation of specific GpG sites located within the *ABI3* promoter [2]. These findings suggested that A*BI3* act as a tumor suppressor gene.

In addition to its suppressive role in thyroid cancer, it has been demonstrated that *ABI3* expression is frequently lost in invasive cancer cell lines. Overexpression of *ABI3* in metastatic glioma cells decreased cell motility and the metastasis potential *in vivo* [3]. It was also demonstrated that *ABI3* might regulate the remodeling of the actin cytoskeleton [4–6]. Moreover, DNA methylation and transcriptional silencing of *ABI3* was associated with poor-prognosis in chronic lymphocytic leukemia samples [7].

While the study of the role of ABI3 in cancer has been intensified, the precise mechanism by which ABI3 exerts its tumor-suppressive effect and/or cytoskeleton remodeling in thyroid cells is still unknown.

ABI3 belongs to ABL-interactor (ABI) family proteins, which also includes ABI1 and ABI2. ABI proteins are a class of cytoplasmic molecular adaptors, originally identified as binding partners for the Abl kinases, which contain an amino-terminal homeobox homologous region (HHR), a proline-rich (PR) region and a carboxy-terminal Src-homology 3 (SH3) domain. The interaction between ABL and ABI proteins seems to involve multiple domains, through which they regulate the ABL kinase activity [8].

Up to now, most of studies have focused on the role of ABI1 and ABI2 family proteins. It has been shown that ABI1 or ABI2 proteins are one of the components of a macromolecular complex referred as WAVE regulatory complex (WRC). The WRC consist of five-subunit protein that includes ABl (ABI1, ABI2 or ABI3), WAVE (WAVE1, WAVE2 or WAVE3), Nap1 (also know as NCKAP1), Sra1 (also know as Cyfip1), and HSCP300 [9]. This heteropentameric complex can be assembled from combinations of different isoforms [9–13].

It has been show that ABI1 forms a large protein complex including WAVE2, Nap1, Sra1 and HSCP300 [14]. ABI1 interacts directly with WAVE2 and Nap1 and couples Abl to WAVE2 in response to signaling. The authors also demonstrated that Abl-mediated WAVE2 tyrosine phosphorylation is required for actin polymerization and remodeling [15].

On the other hand, ABI2 forms a trimer with WAVE1 and HSCP300. The two large subunits Sra1 and Nap1 form a platform for the WAVE1/ABI2/HSCP300 trimer. Thus, the proteins are assembled into a mini WRC pentamer [16, 17].

It has been suggested that the WRC is maintained in an inactive state and disruption of this interface resulted in constitutively active complex.

Although ABI3 was recently identified as a component of the WRC, it was suggested that it is functionally distinct from ABI1-WAVE complex as it neither binds to c-ABL nor promotes ABL-mediated phosphorylation of WAVE2 [18].

As each component of the WRC complex appears exchangeable, identifying members that directly interact with ABI3 and the pathway that may control these interactions can provide an important framework for understating the role of ABI3 in tumor initiation and progression.

Here, using proteome-profiling array, we found that the tumor suppressive effects of ABI3 reduces phosphorylation of AKT and GSKβ. Furthermore, immunoprecipitation combined with mass spectrometry studies demonstrates that ABI3 interacts with WAVE2 and CYFIP1, members of WRC. Mutation at residue S342 of ABI3 analysis suggested and pharmacological inhibition of the PI3K/AKT pathway (LY294002) suggested that ABI3 might be phosphorylated by PI3K/AKT at S342. Moreover, our findings suggested that that the non-phosphorylated form of ABI3 seems to be preferentially present in the protein complex. All together, ours results suggests that the molecular axis consisting of ABI3/WAVE2/CYFIP1 might be negatively regulated by PI3K/AKT pathway in thyroid cells.

## RESULTS

### ABI3 is expressed as phosphorylated and non-phosphorylated forms

**Figure 1:**
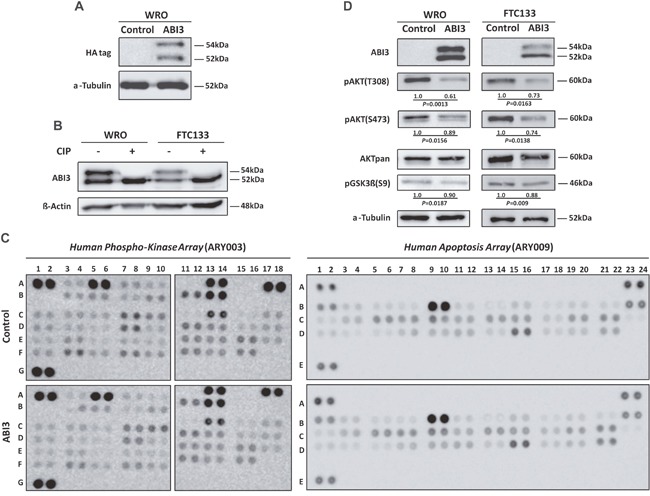
**(A)** Western blot analysis of WRO cells permanently transfected with HA-phCMV2-ABI3 (HA-ABI3) or HA-phCMV2 empty vector (control cells). Two bands were detected: a band at a molecular weight (MW) of ∼54kDa, and a lower band at the expected size of ∼52kDa. **(B)** CIP treatment of the WRO and FTC133 cellular lysates. The upper band disappeared upon CIP treatment in both WRO and FTC133 cells, suggesting that the lower bands correspond to non-phosphorylated forms of ABI3, respectively **(C)** Representative data from Phospho-kinase (ARY003) and apoptosis (ARY009) array analysis. Membranes were incubated with WRO cells expressing ABI3 and control. Proteins were either up-regulated (≥ 1.2, ABI3 expressing cells to control) or down-regulated (≤ 0.8, control to ABI3 expressing cells). **(D)** Western blot analysis of proteins found down-regulated in the Phospho-kinase array assay. The WRO and FTC133 cells expressing ABI3 diminished expression of phospho AKT (pAKT) at both T308/S473 and pGSK3β at S9. Numbers represents the mean obtained from independent experiments.

For functional analysis, a thyroid follicular carcinoma cell line (WRO) was permanently transfected with phCMV2 expressing the full-length HA-tagged ABI3 (HA-phCMV2-ABI3) or with the control vector (HA-phCMV2). Western blot analyses with anti-HA antibody confirmed the expression of the HA-tagged ABI3 protein in WRO cells (Figure 1A). Interestingly, two bands were detected: a band at a molecular weight (MW) of ∼54kDa, and a lower band at the expected size of ∼52kDa. The presence of two bands was also observed when an antibody against ABI3 was tested in both WRO and FTC133 cells transected with the HA-tagged ABI3 expressing construct. These results not only confirm the presence of two forms of ABI3 but also suggest that the upper band is most likely a phosphorylated form of ABI3 (Figure 1B).

The putative phosphorylation event was assessed by treatment of the protein extract with phosphatase (CIP), which catalyzes the removal of phosphate groups from proteins and, consequently, is expected to reverse the mobility shift in the gel. Western blot analysis showed that the upper band disappeared upon CIP treatment in both WRO and FTC133 cells. These results indicate that upper and lower bands correspond to the phosphorylated and non-phosphorylated forms of ABI3, respectively (Figure 1B). Although it has been previously predicted that ABI3 is phosphorylated, our results demonstrate, for the first time, that ABI3 is a phosphoprotein.

### Antibody array proteomic analysis shows that ABI3 regulates distinct cancer-related pathways

It is well known that posttranslational modifications, such as phosphorylation, are tightly regulated and play significant roles in regulating signaling transduction pathways. As phosphorylation can rapidly turn on and off signaling events, we used an antibody array for the simultaneous measurement of relative levels of phosphorylation of 46 intracellular serine/threonine/tyrosine kinases. We additionally assessed the relative expression levels of 35 apoptosis-related proteins. We considered differentially expressed those with *p*-value (*P*<0.05). As even very small fold-changes might be considered statistically significant, we also ranked significant proteins by fold-change. A cut-off of at least 20% difference (<0.80 for down-regulation or >1.2 for up-regulation) between the 2 biological groups (ABI3 expressing cells vs controls) was used. (Supplementary Table 1). Considering both *p*-value and fold-change criteria simultaneously, nearly 26 proteins were down-regulated in WRO cells expressing ABI3. Intriguingly, phosphorylation of key components of the PI3K/AKT (AKT and GSK3α/β), AMPK (AMPKα1 and AMPKα2), mTOR (TOR and p70S6 Kinase), p38 MAPK (p38α, MSK1/2 and HSP27) and SAPK/JNK (JNK and c-Jun) pathways were down-regulated following ABI3 expression. Additionally, ABI3 reduces the phosphorylation of Src family of cytoplasmic tyrosine kinases (Src, Lyn, Lck, Fyn, Yes, Fgr, Pyk2, Hck and FAK), signal transducer and activator of transcription factors (STAT2 and STAT5B) and PLCγ-1 proteins. The expression of β-Catenin and XIAP proteins were significantly increased in WRO cell expressing ABI3 (Supplementary Table 1).

We next applied Kinase Enrichment Analysis (KEA) (adjusted *P*<0.05), an alternative approach to recognize signaling pathways activated following ABI3 expression. The list of the genes considered statistically significant and reported in Supplementary Table 1 (*p*-value and fold-change criteria simultaneously) was used as input for computing enrichment with existing lists created from prior knowledge organized into gene-set libraries [9].

**Figure 2:**
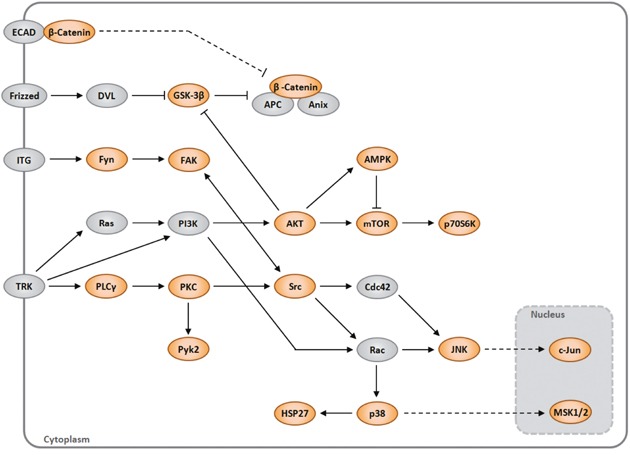
A network map portraying the relationships among the proteins identified as modulated by ABI3 (orange) in the context of cell signaling pathways The gray proteins were not evaluated in array.

KEA ranked *GSK3*β, *GSK3*α, p70S6 Kinase, *SRC*, *AKT1*, *HSP27*, *CTNNB1*, *XIAP*, *PLCγ1* and *TOR* proteins (*P*=0.021, Z-score=-1,062), are most significantly associated. Additionally used the Protein Analysis Through Evolutionary Relationships (Panther). This approach demonstrated enrichment for the RAS pathway (*P*=0.0009, Z-score=-1,398) (Gene list: *JNK1/2, GSK3*β, *JUN, GSK3*α, *AKT1 and p38*α) (P<0.0001). A network map portraying the relationships among these proteins in the context of cell signaling pathways is shown (Figure 2).

### ABI3 diminished phosphorylation of PI3K substrates

As PI3K substrates are enriched based on KEA analysis, we next validated the expression of key components of PI3K pathway by western blotting analysis. The forced expression of ABI3 in two follicular thyroid carcinoma cell lines (WRO and FTC 133) markedly decreased the phosphorylation of AKT at both T308 and S473, as well as the phosphorylation of the downstream-targeted protein pGSK3β at S9 (*P*<0.05) (Figure 1D).

### WAVE 2 and CYFIP1 are ABI3-interacting proteins

We next sought to identify ABI3-interacting proteins that may be involved in modulating or even integrating signaling pathways found modulated by antibody proteome array. Total protein extract from WRO cells transfected with constructs expressing HA-tagged ABI3 was immunoprecipitated with anti-HA antibody, henceforward named HA-IP. To distinguish the irrelevant background proteins, HA-GFP (Green Fluorescent Protein) was used as negative control. To determine the identity of co-immunoprecipitated proteins, three independent HA-IP and HA-GFP were submitted to mass spectrometry (MS) analysis. This analysis revealed 77 proteins in all IPs. To help to identify the bona fide interacting proteins we initially excluded all proteins that appeared only in the HA-GFP control samples (25 proteins). For the proteins that were identified in both HA-ABI3 and HA-GFP immunoprecipitations we used LFQ (label free quantitation) intensity ratio as a filtering parameter. This led to the exclusion of 11 additional proteins which a HA-ABI3/HA-GFP LFQ intensity ration lower than four-fold. Five other proteins were also excluded on the basis that there was no detectable LFQ intensity associated to the peptides identified for them. By using these criteria, we identified 37 proteins with high probability to be ABI3-binding partners (Supplementary Table 2), including three of the five subunits of the WRC complex (WAVE2, NAP1and CYFIP1). The list also included other proteins that are likely to be involved in the activation of the WAVE complex (FABP5, TUBB3, MYH9 and PDIA3) (Table 1). Moreover, we identified abundant proteins such as actin (ACTBL2), tubulin (TUBA1A) and tropomyosin (TPM1). As WRC controls actin cytosleletal dynamics, it may represent specific protein interactions rather than contaminant proteins.

**Table 1:** List of proteins that are likely to be involved in the activation of the WAVE regulatory complex identified by mass spectrometric analysis

Protein	HA-ABI3/HA-EGFP^b^		
	HA-IP 1^a^	HA-IP 2^a^	HA-IP 3^a^
WAVE2 (WASF2)	2/0	3/0	2/0
CYFIP1	4/0	5/0	8/0
NCKAP1	0/0	1/0	1/0
FABP5	0/0	1/0	1/0
TUBB3	0/0	1/0	1/0
MYH9	14/5	33/24	42/5
PDIA3	2/0	2/3	3/0

^a^HA IP: HA-ABI3 immunoprecipitation.

^b^Unique Peptides.

**Figure 3:**
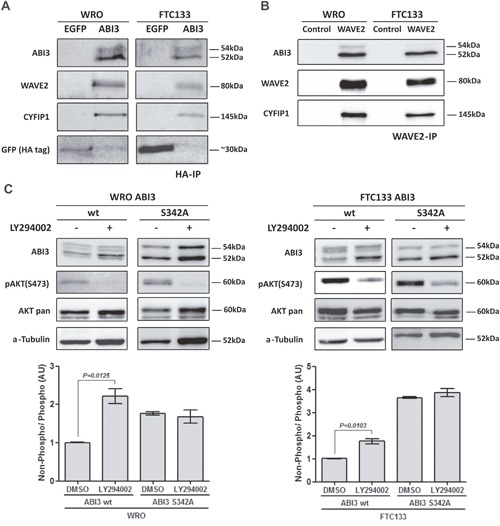
**(A)** ABI3 co-immunoprecipitated with WAVE2 and CYFIP1 in WRO and FTC133 cells evaluated by HA immunoprecipitation. **(B)** WAVE2 immunoprecipitation (WAVE2-IP) confirms that WAVE2, ABI3 and CYFIP1 participate in the same protein complex. The lower band of ABI3 preferentially co-Immunoprecipitates with the protein complex. **(C)** LY294002 treatment showing that the inhibition of AKT pathway concurrently increased the levels of non-phosphorylated ABI3 (lower band). The same increased in the levels of non-phosphorylated ABI3 was observed in ABI3 S342A mutant independently of LY294002 treatment, suggesting that this site was responsible to the modulation observed in ABI3 wt. Values in the graphics represents the mean obtained from independent experiments.

Based in the fact that WAVE2 was identified as putative interacting partner of ABI3 in non-thyroid cells [4] and that WAVE2 and CYFIP1 may participate in the same protein complex, the association of ABI3 with WAVE2 and CYFIP1 was validated by co-immunoprecipitation followed by western blot experiments. We found that ectopically expressed ABI3 co-immunoprecipitated with WAVE2 and CYFIP1 in both cell lines (Figure 3A). To further investigate the interplay between ABI3, WAVE2 and CYFIP1, we ectopically expressed the full length HA-tagged ABI3 in WRO and FTC133 cells and performed IP using the anti-WAVE2 antibody (WAVE2-IP). ABI3 and CYFIP1 co-immunoprecipitated with WAVE2 in both WRO and FTC133 cells, suggesting that these proteins physically interact (Figure 3B). Interestingly, when cell lysate extracted from WRO and FTC133 cells were probed with anti-WAVE2 antibody, the non-phosphorylated form of ABI3 (lower migration protein band) preferentially co-precipitated with WAVE 2 in both cell lines (Figure 3B). These findings may suggest that phosphorylation of ABI3 may generate an inactive form of ABI3 that is unable to interact withWAVE2.

### Mutation of ABI3 at residue S342A affects the expression of phosphorylated form of ABI3

Once CIP treatment indicates that ABI3 is a phosphoprotein (Figure 1B), ABI3 ectopic expression significantly inhibited AKT/GSK3β phosphorylation in thyroid carcinoma cells (Figure 1C and 1D), and phosphorylated form of ABI3 might represent an inactive form of the protein (Figure 3B), we next searched for putative phosphorylation sites using *in silico* analysis and data available from literature. Databases (PhosphoSite Plus, UniProt and HPRD) were used to search for the exact position of known as well as putative phosphorylation sites. We additionally used Scansite3 to identify which families of kinase would be most likely to phosphorylate a given substrate. Twelve phosphorylation sites were identified in the PhosphoSite Plus database (Supplementary Table 3). ABI3 has been previously reported to be phosphorylated at S213, S216 and Y341by mass spectrometry analysis [17, 19–21]. Proteomic and phosphoproteomic characterization also showed that ABI3 was phosphorylated at S342 in mouse and human tissues [17, 19–21].

Although these analyses demonstrated that ABI3 is a target of multiple phosphorylations, for most of them, the biological significance is largely unknown. Interestingly, the Scansite3 predicts T165 and S342 are potential phosphorylation sites for AKT and S202 is a potential phosphorylation site for GSK3β.

As the ABI3 residue S342 is located within SH3 domain, which is one of the most well-known protein domains associated with signaling involved in most basic cellular processes as well as in pathological conditions such malignant transformation, and the conservation of phosphosites has been used to underscore sites that are more likely to be functionally relevant, we next investigated whether S342 is highly conserved in other species such as *Macaca mulatta, Canis lupus familiaris*, *Bos Taurus*, *Mus musculus* and *Rattus norvegicus*. Using phosphosite, the S342 is extremely conserved (100%) among species examined, which further confirm that this site is likely to be involved in related signaling networks with functionally important roles (Supplementary Figure 1).

As an initial attempt to determine whether the S342 is critical for the ABI3 phosphorylation, we constructed an ABI3 mutant clone in which the candidate Serine was replaced to Alanine (S342A). WRO cells expressing the ABI3-S342A mutant preferentially expressed the non-phosphorylated form of ABI3 (Figure 3C, *P*<0.05).

Moreover, cells were treated with LY294002, a PI3K/AKT potent pharmacological inhibitor. As expected, the LY294002 treatment abolishes AKT phosphorylation at S473 in WRO cells and attenuates its phosphorylation in FTC133 cells (Figure 3C). Inhibition of AKT activity was accompanied by a concomitant increase in the expression level of non-phosphorylated form of ABI3 (52 kDa), with respect to its phosphorylated form (54 kDa) (*P*=0.0125; Figure 3C).

The magnitude of the band shift toward the non-phosphorylated form (lower band) in the WRO cells expressing ABI3 Wild type (Wt) following LY294002 treatment is comparable to that seen in ABI3-S342A mutant, independently of the treatment with LY294002. In FTC133 cells the shift toward the non-phosphorylated form was even higher in the ABI3-S342A mutant. These results, graphically represented, may suggest that other protein/signaling pathway might phosphorylate S342 in this cell line (Figure 3C).

### ABI3 may interact with WRC in a PI3K/AKT dependent way

**Figure 4:**
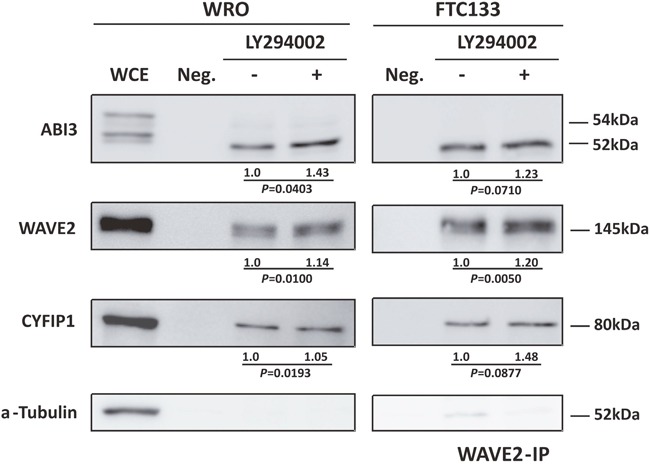
Co-immunoprecipitation of ABI3, WAVE2 and CYFIP1 after LY294002 treatment showing that AKT pathway not only interfere in ABI3 phosphorylation but also in its affinity to WRC complex WCE = Whole Cell Extract. Numbers represents the mean obtained from independent experiments.

As the experimental results presented above suggest that WAVE2 and CYF1P1 directly interact with ABI3 and that PI3K/AKT pathway may regulates the basal activity of ABI3, we next investigate whether PI3K/AKT inhibitors may affect protein interactions. To this, WRO and FTC133 cells ectopically expressing ABI3 were treated with LY294002. The whole-cell extracts (WCE) was immunoprecipitated with anti-WAVE2 antibody and the quantity of ABI3, WAVE2 and CYFIP1 was measured by western-blot. Two independent WAVE2-IP were carried out for each experiment and one representative result is shown in Figure 4. In WRO cells, we observed a significant increase in co-immunoprecipitation of the three proteins (p<0.05) suggesting that inhibition of the PI3K/AKT pathway induced an increased in ABI3 non-phosphorylated form as well as lead to an increase in its affinity to WAVE2 and CYF1P1. Although not statically significant, similar results were observed in FTC133 cells.

### ABI3 expression positively correlates with WAVE2 and CYFIP1 in thyroid tissues

**Figure 5:**
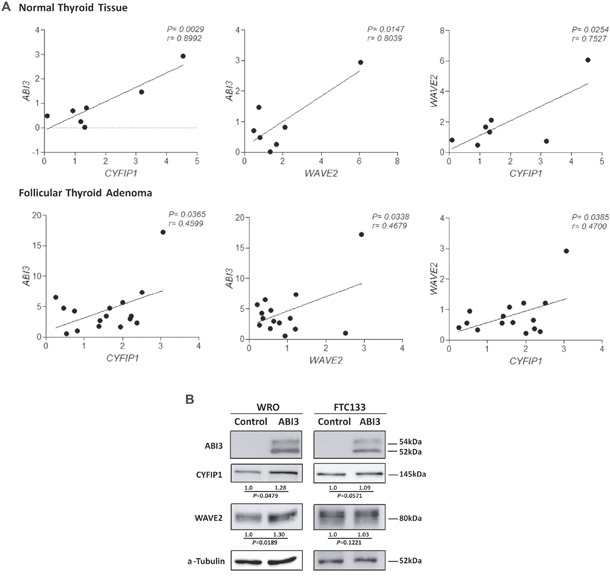
**(A)** Expression of *ABI3*, *WAVE2* and *CYFIP1* in normal thyroid tissue and FTA by quantitative RT-PCR. A significant positive correlation was found between the expression of ABI3, WAVE2 and CYFIP1 in thyroid samples. **(B)** WRO and FTC133 cells ectopically expressing ABI3 also showed high level of WAVE2 and CYFIP1 protein expression. Numbers represents the mean obtained from independent experiments.

We previously demonstrated *ABI3* is expressed in normal thyroid and follicular thyroid adenoma (FTA), while its expression is lost in most follicular thyroid carcinomas. Therefore, to be able to investigate a correlation among ABI3/WAVE2/CYF1P1 expression in thyroid samples, normal thyroid tissues and FTA were chosen to assess the expression of ABI3 WAVE2 and CYF1P1 by qRT-PCR. We observed a significant positive correlation between the mRNA expression of *ABI3*, *WAVE2* and *CYFIP1* in both FTA and normal thyroid tissues (p<0.05) (Figure 5A). As most thyroid carcinomas did not express *ABI3*, the correlation among *ABI3*, *WAVE2* and *CYFIP1* was not performed in this set of samples.

Since a positive correlation was seen in thyroid tissues, the correlation analysis was also performed in follicular thyroid carcinoma cell lines that ectopically expressed *ABI3*. An increase in the levels of the *CYFIP1* and *WAVE2* proteins in WRO cells expressing *ABI3* as compared to control that do not express ABI3 (empty vector) (p<0.05). Although not statically significant, we observed a similar tendency in FTC133 cells (Figure 5B).

## DISCUSSION

We previously showed that ectopic expression of *ABI3* in a follicular thyroid cancer cell line inhibited cell growth, invasiveness and migration *in vitro*, and reduced tumor growth *in vivo* [22]. We also demonstrated that transcriptional silencing of ABI3 in follicular thyroid cancer cells and follicular thyroid tumors occurs via methylation of specific GpG sites located within the *ABI3* promoter [2]. Although our findings provide evidences to support classification of *ABI3* as a bona fide tumor suppressor gene, the precise mechanism by which *ABI3* exerts its function is still unknown.

As far as we know, we report here for the first time that ABI3 can occur in phosphorylated and non-phosphorylated forms. As phosphorylation can either activate or inactivate signaling pathways, we profiled the phospho-kinase screening array to interrogate the signaling pathways that are regulated by ABI3. Our phosphoprotein array analysis shows that re-expression of ABI3 down-regulates the phosphorylation of components of the PI3K/AKT, AMPK, mTOR, p38 MAPK and SAPK/JNK pathways. To illustrate the interconnection between the pathways, the 5-top pathways we manually combined into a single map (Figure 2). Down-regulation of these pathways, known as crucial to tumor growth and differentiation in different tumors subtypes [23–25], can explain, at least in part, the biological effects observed following forced expression of ABI3 in follicular thyroid carcinoma cells [22].

As the most significant enriched pathway was the PI3K/AKT, we next investigated the activity of major downstream effectors of PI3K. ABI3 expression inhibited AKT phosphorylation (S473 and T308), as well as, GSK3β phosphorylation (Ser9).

We also found that ABI3 up-regulates β-Catenin. It was previously reported that PI3K/AKT/GSK3β pathway regulates β-Catenin activity [26] and that down-regulation of β-catenin expression is associated with more aggressive tumor phenotypes [27]. However, additional studies are needed to better clarify whether ABI3 may link PI3K/AKT/GSK3β and β-Catenin pathways in thyroid cells.

Importantly, up-regulation of the PI3K/AKT pathway is an important mechanism associated with pathogenesis, as well as, with the progression of thyroid cancer [25, 28].

To better understand how ABI3 could interact with PI3K/AKT pathway, immunoprecipitation combined with mass spectroscopy analysis was used to pull-down protein complex and identify those that may interact with ABI3. We identified 37 proteins that can potentially associate with ABI3 and form different types of complexes, including components of the canonical WAVE regulatory complex (WRC) such as CYF1P1, WAVE2 and NAP1. Therefore, the ABI3-WAVE2-CYFIP1 interaction was validated by co-immunoprecipitation assays. The three proteins were co-immunoprecipitated by anti-ABI3 or anti-WAVE2 specific antibodies. Collectively, these data corroborate the results obtained with mass spectrometry, and indicate that WAVE2 and CYF1P1 are interacting partners for the ABI3 protein.

We further show that mutation at S342A and LY294002 treatment increases the expression level of non-phosphorylated form of ABI3, suggesting that PI3K/AKT potentially phosphorylates ABI3 at S342. Interestingly, the S342 site is located within the SH3 domain of ABI3. It has been suggested that the SH3 domain and the proline-rich region found in ABI3 are involved in the interaction with others proteins or complexes [8]. In addition, phosphorylation is a mechanism involved in WRC activation and may influence in the complex conformation [15, 16, 29]. However, the phosphorylation pattern of the members of WRC and their activating kinases are largely unknown.

**Figure 6:**
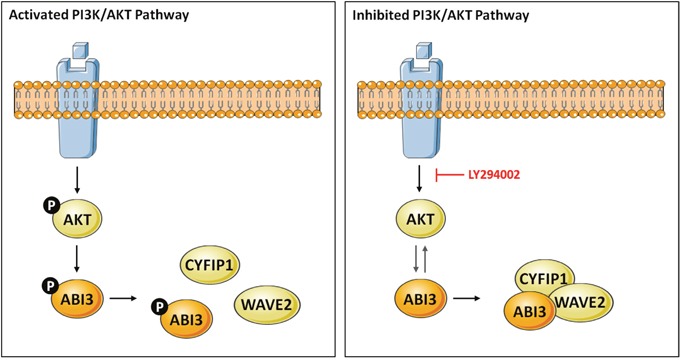
Model describing the putative crosstalk between the PI3K/AKT and WAVE regulatory complex (WRC) in thyroid cells via ABI3 Our data suggests a model whereby PI3K/AKT signaling might control ABI3 phosphorylation at S342. Phosphorylation of ABI3 by AKT inhibits the formation of WRC. In presence of LY294002, a PI3K/AKT inhibitor, the non-phosphorylated form of ABI3 was found within WRC (ABI3, WAVE2 and CYFIP1), confirming the interrelationship between PI3K/AKT and ABI3.

We observed here in immunoprecipitation assays that the non-phosphorylated form of ABI3 is preferentially found in association with the protein complex. Furthermore, our data suggest that AKT may interfere in WRC formation probably by modulation the phosphorylation state of ABI3. We propose that when the AKT pathway is activated, ABI3 is phosphorylated by AKT at residue S342, which inhibits the formation of WRC complex. On the other hand, when the AKT pathway is inhibited with LY294002, ABI3 ceases to be phosphorylated by AKT and the WRC complex can be to be formed (Figure 6).

Remarkably, WRC can be assembled through associations of different isoforms of five proteins: ABI (ABI1, ABI2 and ABI3), Cyfip1/Sra-1 (Cyfip2/PIR121), Nap1 (Hem1), WAVE (WAVE1, WAVE2 and WAVE3) and HSPC300/Brck-1. WRC has been involved in many cellular processes associate with cytoskeleton dynamics including the maintenance of cell shape and morphology, cytokinesis, adhesion, migration, endocytosis and phagocytosis. Dysregulation of WRC components have been associated with cancer development, as well as, metastasis [30–37]. Down-regulation of WAVE2 was associated with a metastatic phenotype in gastric cancer, melanoma cells and adenocarcinoma of the lung [31–33]. Reduced expression of CYFIP1 was also observed during invasion of epithelial tumors, been associated with poor prognosis and appears to cooperate with the oncogene RAS [34]. Interestingly, ABI3 was previously reported to be present in WAVE2 complex in NIH3T3 cells, but the authors demonstrated that ABI3-based WAVE2 complex is functionally distinct from ABI1-based WAVE complex [18]. Other authors did not describe significant changes in the phosphorylation status of either AKT or in MAPK in malignant glioma cells following *ABI3* expression [3]. Importantly, although PI3K is mainly associated with cell growth and proliferation signals downstream from growth factors, it has also been associated with actin cytoskeleton remodeling and this regulation may involve interactions of PI3K and WRC [38]. More precisely, the authors suggested that WAVE, ABI1 and p85 subunit of PI3K might interact to promote actin-polymerization. Therefore, not only the interaction of ABI3 with other canonical components of the WRC may differ slightly according to cell types, but also the mechanism by which ABI3 interferes with components of PI3K/AKT signaling pathways may diverge.

In summary, we here demonstrated that the non-phosphorylated form of ABI3 might be associated with its tumor suppressor effects and also propose a mechanism by which ABI3 links with PI3K/AKT with WRC.

## MATERIALS AND METHODS

Methods are described in greater details in Supplementary data.

### Cell lines

Two follicular thyroid carcinoma cell lines were used. The WRO (UCLA RO-82W-1) was kindly donated by Alfredo Fusco (Facoltà di Medicina e Chirurgia, University Federico II, Naples, Italy). FTC133 was purchased from the European Collection of Authenticated Cell Cultures (ECACC; cat# 94060902). WRO cell have a p53 mutation at codon 223 (P223L). FTC133 has a p53 mutation at codon 273 (R273H). In addition, FTC133 harbor a hemizygous deletion of PTEN with a nonsense point mutation (R130X) in the remaining allele.

### Plasmids constructions and generation of stable cell clones

The full-length cDNA of *ABI3* (accession number NM_016428.2) was cloned into phCMV2 expression vector containing a HA-tag upstream the multiple cloning site using EcoRI/BamHI restriction sites generating phCMV2-ABI3 vector. For generation of HA-GFP-phCMV2 vector, GFP was subcloned from pcDNA-FLAG-GFP. The HA-GFP-phCMV2 and HA-phCMV2 empty vector and were used as negative controls.

Two follicular thyroid carcinoma cell lines, WRO (UCLA RO-82W-1) and FTC133 (ECACC; cat# 94060902) were used. Cells were stably transfected with 10 μg of each construct by electroporation using a Gene Pulser II Electroporation System (Bio-Rad, Hercules, CA). Pools were selected in complete medium supplemented with Geneticin 800 μg/mL (WRO) and 700 μg/mL (FTC133) (Invitrogen, Life Technologies, Grand Island, NY).

### Proteome profiler antibody array

Cell lysates from WRO cells stably transfected with either ABI3 or control (empty vector) were incubated with Human Phospho-Kinase Array Kit (cat. # RY003), which detects relative phosphorylation levels of 46 intracellular serine/threonine/tyrosine kinases, and Human Apoptosis Array Kit (cat# ARY009), which detects levels of 35 apoptosis-related proteins (R&D Systems, Minneapolis, MN). The intensity score of each duplicated spot was measured using ImageQuant LAS4000 Analyzer (GE Healthcare, Chicago, IL) and quantified using ImageQuant TL software (GE Healthcare). The averaged intensity was calculated by subtracting the averaged background signal, according to the manufacturer's instructions. Fold changes were calculated based on the average density values of each protein expressed from WRO ABI3 expressing cells divided by the average density values of control cells.

### Functional enrichment analysis

In order to visualize enriched pathways, the list of proteins that were found differentially expressed in WRO cells expressing ABI3, compared to the control, were upload at Enrichr analysis tool available online (http://amp.pharm.mssm.edu/Enrichr/).

### HA-tag immunoprecipitation (HA-IP) and mass spectrometry analysis

The lysates of WRO cells expressing the HA-tagged proteins (ABI3 and GFP) were incubated at 4°C for overnight with an EZview Red Anti-HA agarose Affinity Gel (Sigma Aldrich, St. Louis, MO) on a rocking platform. The recovered gel was washed three times with wash buffer (50 mM Tris-HCl pH=8.0, 100 mM NaCl). Bound proteins were eluted by competition with a synthetic HA peptide (300 ng/μL) (Sigma Aldrich). The resultant immunoprecipitation eluates were directly analyzed by liquid chromatography coupled with tandem mass spectrometry (LC-MS/MS). Proteins from the HA-ABI3 and HA-GFP pull-downs were denatured, reduced and alkylated. Four micrograms of peptides were analyzed in triplicate by LC-MS/MS in a Thermo Scientific Easy-nLC 1000 system coupled to a LTQ Orbitrap XL ETD (mass spectrometry facility RPT02H PDTIS, Fiocruz Parana). The ten most intense ions were sequentially isolated and fragmented in the linear ion trap using collision-induced dissociation at a target value of 30,000. Peaklist picking, protein identification, quantification, and validation were obtained using the MaxQuant platform (version 1.5.2.8). Antibodies used in this study are detailed in supplementary data (Supplementary Table 4). Proteomic data analysis is described in details in supplementary data.

### Protein G-sepharose immunopreciptation with WAVE2 antibody

For WAVE2 immunoprecipitation (WAVE2-IP), WRO cells expressing the HA-tagged proteins (ABI3 and GFP) were lysate and incubated at 4°C overnight with 1:50 WAVE2 antibody (Supplementary Table 4) on a rocking platform. After antibody incubation, Protein beads were coupled to the extract for 3 h at 4°C in a rocking platform and washed three times with lyses buffer. Complexes were eluted from the beads with 0.1 M glycine-HCl pH 2.2 and neutralized with 1 M Tris-HCl pH=8.0. Eluted proteins were used in western blot analysis.

### Alkaline phosphatase treatment

Cells lysates from WRO and FTC133 cells expressing ABI3 were treated with 1U/μg of protein of Calf Intestinal Alkaline Phosphatase (CIP, New England Biolabs) at 37°C for 1 h. Cell lysates untreated with CIP were used as negative control.

### Thyroid samples

The series consists of 23 thyroid samples (7 normal thyroid tissues and 16 follicular thyroid adenoma) obtained from patients who underwent thyroid surgery at Hospital São Paulo (Universidade Federal de São Paulo), conducted under the approval of the Review Boards and Research Ethical Committee.

Total RNA was isolated from thyroid samples and Cell lines using TRIzol reagent (Invitrogen, Life Technologies, Grand Island, NY). RNA was reverse transcribed into cDNA using Super-Script III Reverse Transcriptase kit with an oligo(dT)12–18 primers. An aliquot (1μL) of cDNA was used in a PCR reaction containing SYBR Green PCR Master Mix (Applied Biosystems, Life Technologies) and 3,2 pmol of each specific primer for the target genes (*ABI3*, *WAVE2* and *CYFIP1*) or reference gene (*RPS8*) (Supplementary Table 5). The relative expression (RE) was calculated according to the comparative ΔΔCt method and used to correlate the expression of ABI3/WAVE2/CYF1P1.

### Analysis of phosphorylation sites in ABI3 and site-directed mutagenesis

A search for putative phosphorylation sites of human ABI3 was performed using ABI3 data registered in the public databases (Supplementary Table 3) and candidates residues were submitted to Motif Scan analysis at Scansite 3 (http://scansite3.mit.edu/). This combined analysis revealed S342 as a likely candidate site for phosphorylation (Supplementary Table 3). To determine whether this residue (S342) is phosphorylated, the candidate serine was mutated to alanine (S342A) using the QuikChange Lightning Site-Directed Mutagenesis Kit (Agilent Technologies, Santa Clara, CA) according to the manufacturer's instructions. The mutant plasmid (phCMV2-ABI3-S342A) was stable transfected into WRO and FTC133 cells and the expressed mutant protein was evaluated by western blot analysis.

### LY294002 treatment

As ABI3 is likely to be phosphorylated by AKT on Serine residue 342, we further evaluate the impact of PI3K/Akt pathway on the phosphorylation of ABI3 using LY294002, a potent inhibitor of PI3K that lead to the dephosphorylation of AKT at both T308 and S473. To this end, WRO and FTC133 cells expressing ABI3 were treated for 2 h with 20 μM of the LY294002 (Cell Signaling Technology). Cells were collected, lysed and submitted to western blot analysis.

### Statistical analysis

Statistical analyses were performed using GraphPad Prism v5.01 Software (GraphPad Software). We used Student t test or Mann-Whitney test. Results were expressed as mean±SD and P<0.05 was considered statistically significant.

## SUPPLEMENTARY FIGURE AND TABLES




