# Supplementary material for: ABI3, a component of the WAVE2 complex, is potentially regulated by PI3K/AKT pathway
Source: Oncotarget. 2017 Jun 29;8(40):67769–81. doi: 10.18632/oncotarget.18840 (PMC5620210; doi:10.18632/oncotarget.18840)
Supplement: Supplementary file 1 [file oncotarget-08-67769-s001.pdf]

ABI3, a component of the WAVE2 complex, is potentially regulated by PI3K/AKT pathway

SUPPLEMENTARY MATERIALS

Plasmids constructions

The full length cDNA of *ABI3* (accession number NM_016428.2) was synthetized from a RNA isolated from a FTA sample using using Super-Script III Reverse Transcriptase kit with an oligo(dT)$_{12–18}$ primer and 10 units of RNase inhibitor (Invitrogen, Life Technologies, Grand Island, NY), according to the manufacturers' recommendations. To insert *EcoR*1 and *BamHI* restriction sites at the 5’ and 3’-terminii of the cDNA products, a sense primer containing an *Eco*RI (underlined) and an antisense primer with a *Bam*HI restriction site (underlined) were used to generate the ABI3 cDNA (Supplementary Table 5). An aliquot of the cDNA was used as template in a 50-µL PCR reaction containing 0.8 mM dNTPs, 2 mM $MgSO_4$, 60 mM Tris–SO_4 (pH 8.9), 18 mM (NH4)2SO4, 1 U Platinum Taq DNA Polymerase High Fidelity (Invitrogen) and 10 pmol of each sense and antisense primers. The amplified product was purified and digested with restriction endonucleases *Eco*RI and *Bam*HI (New England Biolabs, Hitchin, UK). The PCR fragment was cloned into phCMV2 expression vector containing a HA-tag upstream the multiple cloning site using EcoRI/BamHI restriction sites generating the plasmid HA-ABI3. Ligation was performed using a 1:30 molar ratio of vector to insert and T4 DNA Ligase (New England Biolabs), following the manufacturer’s instructions. Ligation mixtures (5 µL) were then transformed into a 50-µL aliquot of chemically competent Mach1 cells (Invitrogen) using the heat shock method. Transformed bacteria were spread on LB-ampicillin (100µg/mL) plates and single colonies were isolated. Following overnight growth, plasmids were purified using PureLink HiPure Plasmid Midiprep Kit (Invitrogen). The construct was fully sequenced using the BigDye Terminator v3.1 Cycle Sequencing Kit.

To generate the HA-GFP construct (control vector), both pcDNA-FLAG-GFP [1] and HA-phCMV2 expression vectors were used. Precisely, the pcDNA-FLAG-GFP and HA-phCMV2 expression vectors were digested with *Eco*RI restriction enzyme. The GFP cassette was gel-purified and inserted at the 3’ end of the HA coding sequence, using *Eco*RI cloning site of vector. Insertion of the GFP cassette was confirmed by *Eco*RI digestion.

Generation of stable cell clones

Two follicular thyroid carcinoma cell lines were used. The WRO (UCLA RO-82W-1) was kindly donated by Alfredo Fusco (Facoltà di Medicina e Chirurgia, University Federico II, Naples, Italy). FTC133 was purchased from the European Collection of Authenticated Cell Cultures (ECACC; cat# 94060902). WRO cells were cultured in DMEM and FTC133 in DMEM:F12 (vo/vol) supplemented with 10% (vol/vol) fetal bovine serum, 100 U/mL of penicillin and 100 µg/mL of streptomycin (Gibco, Life Technologies). Cells were maintained at 37°C in a 5% CO2 humidified atmosphere. Cells were stably transfected with 10 µg of each construct encoding *ABI3* gene (HA-ABI3) and controls (HA-phCMV2 and HA-GFP-phCMV2) by electroporation using a Gene Pulser II Electroporation System (Bio-Rad, Hercules, CA), as described [2]}. Pools were selected in complete medium supplemented with Geneticin 800 µg/mL (WRO) and 700 µg/mL (FTC133). The stable expression of the different constructs was confirmed by Western Blot analysis of the target protein. At least two independent experiments were performed.

Preparation of protein lysates and western blot analysis

Cellular protein lysates were collected under cell exponential growth. Where indicated, cells have been also treated with LY294002 (Cell Signaling Technology). Cells were harvested on ice after washing with cold PBS and were lysed with Triton lysis buffer (1% Triton, 20 mM Tris-HCl pH 7.5, 130 mM NaCl, 2.5 mM sodium phosphate, 10 mM NaF and 1mM $NaVO_4$) supplemented with a Protease Inhibitor Cocktail Tablets (complete, Roche, New York, NY). The lysates were clarified by centrifugation at 8.000 g for 10 min at 4°C. Total protein concentration was determined using the BCA method (Thermo Scientific, San Jose, CA). For immunoblot analysis proteins (25 µg) was resolved on 8-12% SDS-PAGE and transferred to nitrocellulose membranes (Bio-Rad). Subsequently, the membranes were blocked with 5% not fat dried milk in 1X TBS 0.1% Tween-20 and

incubated at 4°C for at least 16 hours with the indicate antibodies (Supplementary Table 2). Membranes were incubated with Pierce ECL Western Blotting Substrate (Thermo Scientific, San Jose, CA) or Immobilon Western Chemiluminescent HRP Substrate (Merck Millipore, Darmstadt, GE) when we needed to detect lower levels of proteins. The chemiluminescent signals were detected by ImageQuant LAS4000 Analyzer (GE Healthcare, Chicago, IL). At least two experiments were performed. The band intensities were quantified using the ImageQuant TL software (GE Healthcare) and corrected for background differences. After background correction, the data were normalized to the expression of β-actin or α-tubulin (loading control) and expressed as fold-change.

Proteome profiler antibody array

Human Phospho-Kinase Array Kit and Human Apoptosis Array Kit (cat. # RY003), which detects relative phosphorylation levels of 46 intracellular serine/threonine/tyrosine kinases, and Human Apoptosis Array Kit (cat# ARY009) which detects levels of 35 apoptosis-related proteins, were purchased from R&D Systems (Minneapolis, MN). WRO cells were stably transfected with either ABI3 or control (empty vector), as described above. Cells were lysed with Lysis Buffer 6 (R&D Systems). Cell lysates were clarified by microcentrifugation at 14,000 rpm for 5 min, and the supernatants were subjected to protein assay. Membranes were incubated with 300 µg (ARY003) or 400 µg (ARY009) of cellular extract for 16 hours at 4°C, on a rocking platform. The membranes were washed twice with wash buffer (R&D Systems) and then were incubated with Chemiluminescent reagent (R&D Systems). Array images were analyzed using ImageQuant LAS4000 Analyzer (GE Healthcare, Chicago, IL) and quantified using ImageQuant TL software (GE Healthcare), as described above. Each spot was detracted by the averaged background level from negative control spots. The expression values were calculated as mean of the duplicated spots, representing each protein, normalized by the density levels of the positive control spots present on the membrane. for positive control present in the membrane. The fold change was calculated as the expression observed in the experimental membrane (WRO expressing ABI3) relative to the control membrane (WRO expressing empty vector). The fold changes were determined following two criteria: 1) a protein expression ratio of the control expressing cells to the ABI3 expressing cells ≤0.8 was considered down-regulation and 2) a protein expression ratio of the ABI3 expressing cells to the control cells ≥1.2 was considered up-regulation. The list of antibodies and their position on the arrays can be found at http://www.rndsystems.com/pdf/ARY003.pdf and http://www.rndsystems.com/pdf/ARY009.pdf.

Functional enrichment analysis

In order to visualize enriched pathways, the list of proteins that were found differentially expressed in WRO cells expressing ABI3, compared to the control, were upload at Enrichr analysis tool available online (http://amp.pharm.mssm.edu/Enrichr/).

HA-tag immunoprecipitation (HA-IP) and mass spectrometry analysis

WRO cells expressing the HA-tagged proteins (ABI3 and GFP) were washed harvested by scrapping on using cold PBS and were lysed using cold lyses buffer (50 mM Tris-HCl pH 8.0, 100 mM NaCl, 10 mM NaF, 1mM $NaVO_4$, 0.4% n-Octyl-β-D-glucoside) supplemented with 2X Protease Inhibitor Cocktail Tablets (Complete, Roche). The lysates were centrifuged at 8.000 g for 10 min at 4°C. The supernatants were collected and were incubated at at 4°C for overnight with an EZview Red Anti-HA agarose Affinity Gel (Sigma Aldrich, St. Louis, MO) on a rocking platform. The recovered gel was washed three times with wash buffer (50 mM Tris-HCl pH=8.0, 100 mM NaCl) and the bound proteins were eluted by competition with an HA peptide (300 ng/µL) (Sigma Aldrich) on a rocking platform at 4°C for 2 h. The resultant immunoprecipitation eluates were directly analyzed by liquid chromatography coupled with tandem mass spectrometry (LC-MS/MS), as described below.

Protein digestion and LC-MS/MS analysis

Proteins from the HA-ABI3 and HA-GFP pull-downs were denatured in 6 M urea, 2 M thiourea, 10 mM HEPES, reduced with 1 mM DTT and alkylated with 5.5 mM iodacetamide. The samples were diluted 1:5 with 50 mM ammonium bicarbonate and digested with trypsin (Promega) using a 1:50 mass ratio. The samples were acidified with 0.5% trifluoroacetic acid and desalted on C18 spin columns. Four micrograms of peptides were analyzed in triplicate by LC-MS/MS in a Thermo Scientific Easy-nLC 1000 system coupled to a LTQ Orbitrap XL ETD (mass spectrometry facility RPT02H PDTIS - Carlos Chagas Institute - FIOCRUZ Parana). Peptide separation was carried out on a 15 cm microcapillary column (75 µm I.D.; fused silica packed with reversed-phase C18, 3 µm resin). Chromatography runs were performed in a flow rate of 250 nL/min from 5 to 40% MeCN, 0.1% formic acid in a 120 min gradient. The mass spectrometer operated in a data-dependent mode to automatically switch between MS and MS/MS (MS^2) acquisition. Survey full-scan MS spectra (at 350 – 1,650 *m/z* range) were acquired in the Orbitrap analyzer with resolution of 60,000 at *m/z* 400 (after accumulation to a target value of 500,000 in the C-trap). The ten most

intense ions were sequentially isolated and fragmented in the linear ion trap using collision-induced dissociation at a target value of 30,000. The "lock mass" option was set to 445,120025 *m/z*, enabling in all full scans an improvement of precursor ions mass accuracy [3].

Proteomic data analysis

Peaklist picking, protein identification, quantification, and validation were obtained using the MaxQuant platform (version 1.5.2.8) [4], which includes the algorithm Andromeda [5] for database searching. Default parameters of the software were used for all analysis steps, unless stated otherwise. Proteins were searched against a "decoy database" prepared by reversing the sequence of each entry of the used database and appending them to the forward sequences. The search was conducted against a human protein sequence database (containing 70,071 protein sequences, updated in August 09, 2015) and the green fluorescent protein sequence both from the UNIPROT database. This database was complemented with frequently observed contaminants (porcine trypsin, *Achromobacter lyticus* lysyl endopeptidase, and human keratins) and their reversed sequences. Search parameters specified a MS tolerance of 4.5 ppm, a MS/MS tolerance of 0.5 Da, and full trypsin specificity, allowing for up to two missed cleavages. Carbamidomethylation of cysteine was set as a fixed modification. Oxidation of methionine, and N-terminal acetylation (protein) were allowed as variable modifications. For validation of the identifications, a minimum of seven amino acids for peptide was required. In addition, a false discovery rate (FDR) threshold of 0.01 was applied at both peptide and protein levels. The option match between runs was enabled to increase protein identification. Protein quantification was performed using a label-free (LFQ) approach, where peptides eluting from each LC run are detected as three-dimensional features-retention time versus signal intensity (extracted ion chromatogram, XIC) versus mass/charge-aligned and compared across runs, as previously describe [6].

Protein G-sepharose immunopreciptation with WAVE2 antibody (WAVE 2-IP)

WRO cells expressing the HA-tagged proteins (ABI3 and GFP) were harvested on ice after washing with cold PBS and were lysed in the lyses buffer (50 mM Tris-HCl pH 8.0, 100 mM NaCl, 10 mM NaF, 1mM $NaVO_4$, 0.4% n-Octyl-β-D-glucoside) supplemented with 2X Protease Inhibitor Cocktail Tablets (Complete, Roche). To reduce nonspecific binding to the Protein G-sepharose beads, the cells lysates were pre-cleared by incubation with rec-Protein G-Sepharose 4B (Invitrogen) for 30 min at 4°C. Subsequently, the supernatants were incubated at 4°C overnight with 1:50 WAVE2 antibody (Supplementary Table 4) on a rocking platform. As negative control, cells lysates were incubated without WAVE2 antibody. After antibody incubation, Protein G- sepharose beads were coupled to the extract for 3h at 4°C in a rocking platform and washed three times with lyses buffer. Complexes were eluted from the beads with 0.1 M glycine-HCl pH 2.2 and neutralized with 1 M Tris-HCl pH=8.0. Eluted proteins were stored at -80°C and Western blot analysis was performed as described above.

Alkaline phosphatase treatment

Under normal conditions ABI3 runs as doublet (a lower band of 52kDa an upper band of 54kDa) in both WRO and FTC1333 cells (Figure 1A and 1C). To determine whether the gel shift of *ABI3* (upper band) is due to its phosphorylation, cells lysates were treated with Calf Intestinal Alkaline Phosphatase (CIP, New England Biolabs). WRO and FTC133 cells expressing ABI3 were lysed in an EDTA-free lyses buffer (50 mM Tris-HCl pH 7.5, 150 mM NaCl, 10 mM NaF, 0.1mM NaVO4, 1% Triton, 10 µg/mL Aprotinin, 10 µg/mL Leupeptin and 10 µg/mL Pepstatin). Next, cell lysates were diluted 1:3 and incubated with CIP (1U/µg of protein) at 37°C for 1h. Cell lysates untreated with CIP were used as negative control.

Thyroid samples

The series consists of 23 thyroid samples (7 normal thyroid tissues and 16 follicular thyroid adenoma) obtained from patients who underwent thyroid surgery at Hospital São Paulo (Universidade Federal de São Paulo). The study was conducted under the approval of the Review Boards and Research Ethical Committee of the Universidade Federal de São Paulo (Process 1278/10).

RNA isolation, cDNA synthesis and quantitative RT-PCR (qPCR)

Total RNA was isolated from thyroid samples and Cell lines using TRIzol reagent (Invitrogen, Life Technologies, Grand Island, NY) according to the manufacturer's recommendation. Complete homogenization was obtained by passing the lysate through a 23-gauge needle. RNA was quantified using a NanoDrop2000c spectrophotometer. Total RNA (1µg) was treated with DNAse (Ambion, Life Technologies), reverse transcribed into cDNA using Super-Script III Reverse Transcriptase kit with an oligo$(dT)_{12-18}$ primer and 10 units of RNase inhibitor (Invitrogen), according to the manufacturers' recommendations. An aliquot (1µL) of cDNA was used in a 12 µL PCR reaction containing SYBR Green PCR Master Mix (Applied Biosystems, Life Technologies) and 3,2 pmol of each specific primer for the

target genes (*ABI3, WAVE2 and CYFIP1*) or reference gene (*RPS8*). The PCR reactions were performed in triplicate, and the Ct was obtained using Applied Biosystem software and averaged (SD < 1.0). The relative expression (RE) was calculated according to the comparative ΔΔCt method. Normal thyroid, vehicle-treated control cells or empty vector transfected cells were used as the control group for ΔΔCt calculation Primers and conditions for PCR are summarized in Supplementary Table 5.

Analysis of candidate phosphorylation sites in ABI3

A search for putative phosphorylation sites of human ABI3 was performed using ABI3 data registered in the public databases such as PhosphoSite Plus (http://www.phosphosite.org/), UniProt (http://uniprot.org/) and Human Protein Reference Database (HPRD, http://hprd.org/). Residue numbers were defined according to human isoform 1 (NP_057512.1). To further evaluate whether the residues identified as potentially phosphorylated could be, phosphorylated by the protein kinases found modulated in the proteome antibody array analysis, we submitted the amino-acid sequence of ABI3 to Motif Scan analysis at Scansite 3 (http://scansite3.mit.edu/). This combined analysis revealed S342 as a likely candidate site for phosphorylation.

ABI3 site-directed mutagenesis

To determine if the phosphorylation at serine 342 (S242), we mutated the serine amino acid codon to alanine (S342A) The phCMV2-ABI3 vector was submitted to site-directed mutagenesis with primers for S342A conversion (Supplementary Table 5) using the QuikChange Lightning Site-Directed Mutagenesis Kit (Agilent Technologies, Santa Clara, CA) according to the manufacturer's instructions. The S342A mutation was confirmed by sequencing. The phCMV2-ABI3-S342A vector was stable transfected into WRO and FTC133 cells and the expressed mutant protein was evaluated by western blot analysis

LY4029002 treatment

The LY294002 (Cell Signaling Technology) PI3-kinase specific inhibitor was employed to test the ABI3 dependence on AKT pathway. About 3.5 x 10^5 WRO and FTC133 cells expressing ABI3 were seeded in 60-mm plates. Cells were starved overnight and pre-treated with 20 µM LY294002 for 2h. As negative control cells were incubated with vehicle (DMSO). Then, FBS was added and cells were collected after 5h and used in western-blot analysis as previously described using. Antibodies against pAKT(S473) and AKTpan were used to determinate the efficiency of LY294002 treatment and ABI3 antibody for ABI3 phosphorylation analysis.

Statistical analysis

Statistical analyses were performed using GraphPad Prism v5.01 Software (GraphPad Software). Comparisons between groups were performed using Student t test or Mann-Whitney test when data did not exhibit a normal distribution verified by Shapiro-Wilk test. Results were expressed as mean±SD and $P<0.05$ was considered statistically significant.

REFERENCES

1. Simabuco FM, Morello LG, Aragao AZ, Paes Leme AF, Zanchin NI. Proteomic characterization of the human FTSJ3 preribosomal complexes. J Proteome Res. 2012;11:3112-26.
2. Latini FR, Hemerly JP, Freitas BC, Oler G, Riggins GJ, Cerutti JM. ABI3 ectopic expression reduces in vitro and in vivo cell growth properties while inducing senescence. BMC Cancer. 2011;11:11.
3. Olsen JV, de Godoy LM, Li G, Macek B, Mortensen P, Pesch R, Makarov A, Lange O, Horning S, Mann M. Parts per million mass accuracy on an Orbitrap mass spectrometer via lock mass injection into a C-trap. Mol Cell Proteomics. 2005;4:2010-21.
4. Cox J, Mann M. MaxQuant enables high peptide identification rates, individualized p.p.b.-range mass accuracies and proteome-wide protein quantification. Nat Biotechnol. 2008;26:1367-72.
5. Cox J, Neuhauser N, Michalski A, Scheltema RA, Olsen JV, Mann M. Andromeda: a peptide search engine integrated into the MaxQuant environment. J Proteome Res. 2011;10:1794-805.
6. Luber CA, Cox J, Lauterbach H, Fancke B, Selbach M, Tschopp J, Akira S, Wiegand M, Hochrein H, O'Keeffe M, Mann M. Quantitative proteomics reveals subset-specific viral recognition in dendritic cells. Immunity. 2010;32:279-89.
7. Huttlin EL, Jedrychowski MP, Elias JE, Goswami T, Rad R, Beausoleil SA, Villen J, Haas W, Sowa ME, Gygi SP. A tissue-specific atlas of mouse protein phosphorylation and expression. Cell. 2010;143:1174-89.
8. Manes NP, Dong L, Zhou W, Du X, Reghu N, Kool AC, Choi D, Bailey CL, Petricoin EF 3rd, Liotta LA, Popov SG. Discovery of mouse spleen signaling responses to anthrax using label-free quantitative phosphoproteomics via mass spectrometry. Mol Cell Proteomics. 2011;10:M110 000927.
9. Han G, Ye M, Liu H, Song C, Sun D, Wu Y, Jiang X, Chen R, Wang C, Wang L, Zou H. Phosphoproteome analysis of human liver tissue by long-gradient nanoflow LC coupled with multiple stage MS analysis. Electrophoresis. 2010;31:1080-9.
10. Raijmakers R, Kraiczek K, de Jong AP, Mohammed S, Heck AJ. Exploring the human leukocyte phosphoproteome using a microfluidic reversed-phase-TiO2-reversed-phase high-performance liquid chromatography phosphochip coupled to a quadrupole time-of-flight mass spectrometer. Anal Chem. 2010;82:824-32.

SUPPLEMENTARY FIGURE AND TABLES

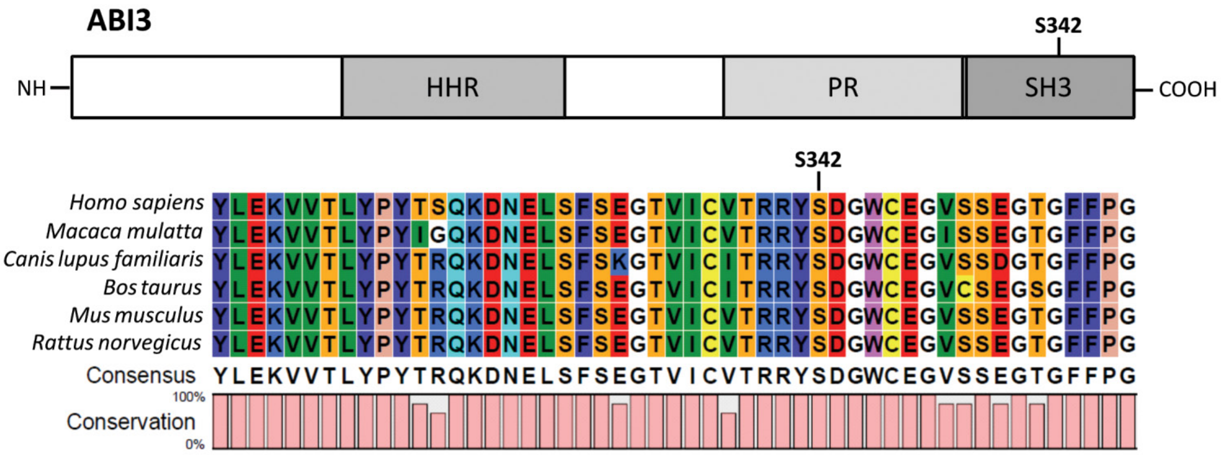

Supplementary Figure 1: S342 site is highly conserved in other species such as *Macaca mulatta, Canis lupus familiaris, Bos Taurus, Mus musculus* and *Rattus norvegicus*.

Supplementary Table 1: List of phosphokinases and apoptosis-related proteins modulated by ABI3

Antibody array	Spot	Protein	Phosphorylation site	Fold-change	Percentage of modulation	*P*-value
ARY003	A3, A4	p38α	T180/Y182	0.58	-42 %	0.0402
ARY003	A7, A8	JNK pan	T183/Y185, T221/Y223	0.39	-61 %	0.0275
ARY003	A9, A10	GSK-3α/β	S21/S9	0.17	-83 %	0.0155
ARY003	B5, B6	MSK1/2	S376/S360	0.67	-33 %	0.0255
ARY003	B7, B8	AMPKα1	T174	0.46	-55 %	0.0235
ARY003	C7, C8	AMPKα2	T172	0.73	-27 %	0.0290
ARY003	B9, B10	Akt	S473	0.60	-40 %	0.0058
ARY003	B11, B12	Akt	T308	0.73	-27 %	0.0028
ARY003	C1, C2	TOR	S2448	0.64	-36 %	0.0324
ARY003	C5, C6	HSP27	S78/S82	0.47	-53 %	0.0105
ARY003	C9, C10	β-Catenin/CTNNB1	-	1.24	+24 %	0.0313
ARY003	C11, C12	p70 S6 Kinase	T389	0.00	-100 %	0.0163
ARY003	D1, D2	Src	Y419	0.66	-34 %	0.0302
ARY003	D3, D4	Lyn	Y397	0.61	-39 %	0.0182
ARY003	D5, D6	Lck	Y394	0.46	-54 %	0.0403
ARY003	D7, D8	STAT2	Y689	0.72	-28 %	0.0115
ARY003	E9, E10	STAT5b	Y699	0.57	-43 %	0.0215
ARY003	D17, D18	PLCγ-1	Y783	0.74	-26 %	0.0349
ARY003	E1, E2	Fyn	Y420	0.74	-26 %	0.0225
ARY003	E3, E4	Yes	Y426	0.57	-43 %	0.0023
ARY003	E5, E6	Fgr	Y412	0.37	-63 %	0.0479
ARY003	E15, E16	c-Jun	S63	0.74	-26 %	0.0434
ARY003	E17, E18	Pyk2	Y402	0.74	-26 %	0.0396
ARY003	F1, F2	Hck	Y411	0.66	-34 %	0.0159
ARY003	F3, F4	Chk-2	T68	0.66	-34 %	0.0262
ARY003	F5, F6	FAK	Y397	0.70	-30 %	0.0294
ARY009	B23, B24	Cytochrome C	-	0.75	-25 %	0.0229
ARY009	D21, D22	XIAP	-	1.55	+55 %	0.0190

Supplementary Table 2: List of proteins identified in the IP of HA-ABI3. The list includes the proteins that were identified in at least two replicates and that showed higher peak intensity relative to the ip with HA-EGFP. Peak intensity was calculated using the label-free quantitation method (lfq) of the maxquant platform.

See Supplementary File 1

Supplementary Table 3: Site-specific phosphorylation of ABI3

Position	Peptide sequence[a]	UniProt (domain)	PhosphoSite plus [b]	HPRD[c]	Motif Scan - scansite 3 (score)	Conservation	Reference
T134	PENLPPLtPYCRRPL	Abi_HHR [d]	H	-	Cdc2 (0.4317), Cdk5 (0.4303) and Erk1 (0.4811)	High/ Medium	-
T165	TQLSRTGtLSRKsIK	Abi_HHR	H/M	-	14-3-3 (0.4218), AKT (0.4741), AMPK (0.6313), CAMKK2 (0.5339), PKCα/β/γ (0.4444), PKCδ (0.4950) and PKA (0.5515)	High	-
S170	TGtLSRKsIKAPATP	Abi_HHR	H	-	Aurora B (0.5966), PKCα/β/γ (0.4541), PKCδ (0.4407), PKCε (0.4208) and PKA (0.5007)	High	-
S203	VVPDGRLSAASSASs	-	M	-	Aurora A (0.5746), Aurora B (0.5598) and GSK3 (0.6035)	High	-
S210	SAASSASsLASAGsA	-	H	-	PKCδ (0.4522)	High	-
S213	SSASsLASAGsAEGV	-	M	-	PDK1 (0.9491)	High	[7, 8]
S216	SsLASAGsAEGVGGA	-	H/M	Phosphorylation	PKCδ (0.4728)	High	[7-9]
T258	EVFQRPPtLEELSPP	Pro-rich [e]	H	-	14-3-3 (0.6000), AMPK (0.7138), ATM (0.4690), DNA PK (0.6222), PKCε(0.5097) and PKA (0.5491)	Low	-
T338	EGTVICVtRRysDGW	SH3 [f]	H	-	-	High	-
Y341	VICVtRRysDGWCEG	SH3	H/M	-	-	High	[7]
S342	ICVtRRysDGWCEGV	SH3	H/M	Phosphorylation	14-3-3 (0.5574), AKT (0.6697), Aurora A (0.2924), Aurora B (0.3321) and PKA (0.4732)	High	[7, 8, 10]
S365	PGNYVEPsC______	SH3	H	-	-	Medium	-

Supplementary Table 4: Antibodies specifications

Antibody	Dilution	Blocking	Cat. number
HA tag	1:1000	5% w/v nonfat dry milk	# 2367 (Cell Signaling Technology)
ABI3	1:500	5% w/v nonfat dry milk	HPA017345 (Sigma Aldrich)
pAKT(S473)	1:1000	5% w/v BSA	# 4070 (Cell Signaling Technology)
pAKT(T308)	1:1000	5% w/v BSA	# 2965 (Cell Signaling Technology)
AKTpan	1:2000	5% w/v BSA	# 4691 (Cell Signaling Technology)
pGSK3β(S9)	1:1000	5% w/v BSA	# 5558 (Cell Signaling Technology)
CYFIP1	1:1000	5% w/v nonfat dry milk	ab108220 (Abcam)
WAVE2	1:1000	5% w/v BSA	# 3659 (Cell Signaling Technology)
β-actin	1:2000	5% w/v BSA	# 4967 (Cell Signaling Technology)
α-tubulin	1:10.000	5% w/v nonfat dry milk	T9026 (Sigma Aldrich)
Anti-Mouse	1:10.000	5% w/v nonfat dry milk	SC2031 (Santa Cruz Biotechnology)
Anti-Rabbit	1:10.000	5% w/v nonfat dry milk	P0448 (Dako)

BSA = bovine serum albumin.

Supplementary Table 5: Primers sequences, PCR conditions and expected product sizes

Gene	Sequence (5' - 3')	Assay	Annealing temp (°C)	Product size (pb)
ABI3	Forward: TAAGCACGCTGGGCCAGAT	Expression	60	166
	Reverse: CAGCCAAAGTTGAGGGGTCT			
WAVE2	Forward: CCCACGTAAAATCAAGACACG	Expression	60	129
	Reverse: AATGCTGCCATTCTGGTACAC			
CYFIP1	Forward: CCATCAGGAAGACCGTGTG	Expression	60	114
	Reverse: CGCGGCGTGGTACTTTTAT			
RPS8	Forward: AACAAGAAATACCGTGCCC	Expression	60	125
	Reverse: GTACGAACCAGCTCGTTATTA			
ABI3	Forward: CCGGAATTCTGATGGCGGAGCTACAGCAGCT	Cloning	60	1250
	Reverse: CCGGGATCCAGGACAGAACACAGCTCACAGC			
ABI3 S342A	Forward: CACTCGCCGCTACGCTGATGGCTGGTGCG	Mutagenesis	55	NA
	Reverse: CGCACCAGCCATCAGCGTAGCGGCGAGTG			

NA = not applicable.
